# Effect of Three Different Preservatives on the Microbiota of Shalgam, a Traditional Lactic Acid Fermented Beverage

**DOI:** 10.3390/foods12224075

**Published:** 2023-11-09

**Authors:** Gamze Nur Mujdeci, Hasan Tanguler, Hasan Macit, Bulent Kabak

**Affiliations:** 1 Department of Food Engineering, Faculty of Engineering, Hitit University, Corum 19030, Turkey; gmujdeci123@gmail.com; 2 Biotechnology Laboratory, Machinery and Manufacturing Technology Application and Research Center, Hitit University, Corum 19030, Turkey; 3 Department of Food Engineering, Faculty of Engineering, Niğde Ömer Halisdemir University, Niğde 51240, Turkey

**Keywords:** lactic acid bacteria, yeast, identification, MALDI-TOF/TOF MS, fermentation, potassium sorbate, sodium benzoate, natamycin, storage

## Abstract

Shalgam is a traditional Turkish beverage derived from the natural fermentation of purple carrots (*Daucus carota*) that boasts valuable antioxidant and prebiotic properties. These features of shalgam increase efforts to enhance its shelf life and ensure safe consumption. In this study, the effects of three different preservatives (sodium benzoate, potassium sorbate, or natamycin) on the physicochemical and microbiological properties of shalgam produced at laboratory scale and stored at room temperature for six months were investigated. Each preservative was used in four different concentrations (25, 100, 400, and 800 mg/L) to assess their impacts on the population of lactic acid bacteria (LAB) and yeast. After determining the total acidity and pH of the samples, colorimetric measurements were performed. The isolated LAB were defined using the matrix-assisted laser desorption ionization time-of-flight mass spectrometry (MALDI-TOF/TOF MS) method. The addition of preservatives did not significantly affect the pH of the shalgam samples (3.44–3.52) compared to the control sample (3.43). However, a slight increase was observed in the total acidity of preservative-treated samples, with the highest level (5.61 g/L lactic acid) recorded in samples containing 100 mg/L sodium benzoate. *Lacticaseibacillus paracasei* subsp. *paracasei*, which has the potential to impart probiotic properties to shalgam, was the predominant LAB species in both non-treated and preservative-treated samples. The use of preservatives significantly reduced the total number of yeasts, which may cause spoilage in shalgam. The results indicate that using sodium benzoate at a concentration of 100 mg/L is the optimum method for shalgam production, resulting in the highest total acidity value obtained. Overall, the findings provide a significant contribution to prolonging the shelf life of shalgam, a beverage with immense production and consumption potential worldwide.

## 1. Introduction

Fermented products obtained via fermentation, which is one of the oldest, most practical, and most economical food storage methods, are consumed with admiration almost all over the world [1]. These include products of commercial importance such as vinegar, wine, beer, table olives, pickles, and dairy products [2]. Additionally, certain lesser-known fermented products, such as boza, tarhana, tempeh, ayran, kefir, hardaliye, gilaburu, and shalgam, are produced in specific regions and cultures [3,4]. Among them, shalgam stands out as a sour, cloudy, and red-purple-colored functional beverage renowned for its high antioxidant content. It has started to be sold in all regions of Turkey, especially in the Southern region, and recently in certain European capitals [3]. 

Shalgam is made from purple (black or red) carrots (*Daucus carota*), baker’s yeast or sourdough, bulgur flour, rock salt, and water. In addition, if available, turnips (turnip radish, *Brassica rapa* L.) are also utilized. The product is obtained through the dual process of lactic acid and ethyl alcohol fermentation [4,5,6]. Shalgam is rich in minerals such as calcium, magnesium, iron, phosphorus, sulfur, iodine, and potassium, as well as vitamins A, B, and C, and phenolic compounds. Shalgam holds significant health-related relevance because of its digestive merits, which are primarily attributed to its inherent probiotic content. This natural probiotic composition contributes to enhanced digestive processes and fosters a robust gut microbiome. Beyond its gastrointestinal benefits, shalgam exerts a multifaceted impact, including cholesterol reduction and relief from constipation [4]. It is very effective at improving bone health by supporting the immune system, increasing iron absorption in the body, protecting cardiovascular health, and maintaining the normal function of the nerves, heart, bones, kidneys, and stomach [7]. 

Nonetheless, the primary challenge for producers lies in the short shelf life of industrially produced shalgam. The product is highly perishable, so immediate consumption is recommended for the best quality and flavor. Although heat treatment can extend the shelf life of shalgam, it is not preferred due to its negative impact on the physical, chemical, and sensory properties of the product [5,6]. It has been reported that heat treatment negatively affects the taste, aroma, anthocyanin content, and color of the product [8]. However, the shelf life of shalgam can be extended up to two years with the addition of preservatives [9].

Chemical preservatives such as sulfites, nitrite and nitrate compounds, benzoic acid, sorbic acid, propionic acid, and their salts, and natamycin play a pivotal role in food preservation, ensuring the safety, quality, and extended shelf life of various food products [10,11]. Among these preservatives, benzoates, sorbates, and natamycin are particularly significant. Benzoates, such as sodium benzoate, are potent antimicrobial agents commonly employed in acidic products like carbonated beverages and fruit-based items. They inhibit the growth of yeasts and molds, thus preventing spoilage. Sorbates, including potassium sorbate, are effective against a broader spectrum of microorganisms, making them suitable for a wide range of acidic and low-pH foods. They act by inhibiting the growth of yeast, mold, and some bacteria [12,13]. Natamycin, a natural antifungal compound, is utilized in dairy products as well as beverages to suppress the growth of undesirable molds and yeasts [14,15]. While the use of benzoic acid-benzoates up to 200 mg/L is legally allowed for shalgam production [16], the use of sorbic acid-sorbates and natamycin is prohibited. 

The quality and shelf life of a product are closely related to its microflora [3]. The dominant microflora in shalgam primarily consists of lactic acid bacteria (LAB) and, to a lesser extent, yeasts [4,9]. However, there are no studies exploring the use of preservatives and the associated changes in microflora, particularly in relation to shalgam preservation. The application of traditional and/or molecular techniques in exploring the impact of preservatives on the microflora of shalgam could offer valuable insights into the changes in microflora composition and enhance our understanding of its shelf life dynamics.

Matrix-assisted laser desorption ionization time-of-flight mass spectrometry (MALDI-TOF/TOF MS) is being used for quick and accurate bacterial identification [17]. This ionization technique facilitates peptide and protein desorption from all cells of cultivated microorganisms, with ions separated based on their molecular mass and charge [18]. MALDI-TOF/TOF MS is a safe and fast method of phenotypic identification that allows bacteria to be profiled by genus, species, and even strain level [17,19].

The aim of this study was to investigate the impact of three different preservatives (sodium benzoate, potassium sorbate, or natamycin) in four different quantities (25, 100, 400, and 800 mg/L) on the microflora of shalgam during six months of storage under room conditions. Following laboratory-scale shalgam production, physicochemical and microbiological analyses were conducted. The LAB isolates were identified using the MALDI TOF/TOF MS method. 

## 2. Materials and Methods

### 2.1. Food Materials

Purple carrots and bulgur flour were obtained from AYRIS Agriculture Food Industry and Trade Company (Adana, Turkey). Baker’s yeast and turnips were purchased from a bazaar in Niğde, Turkey. Sourdough was obtained with the incubation of baker’s yeast at 30 °C for 24 h. Unrefined rock salt was supplied from the local market. Tap water was used in the production of shalgam.

### 2.2. Shalgam Production

**Figure 1:**
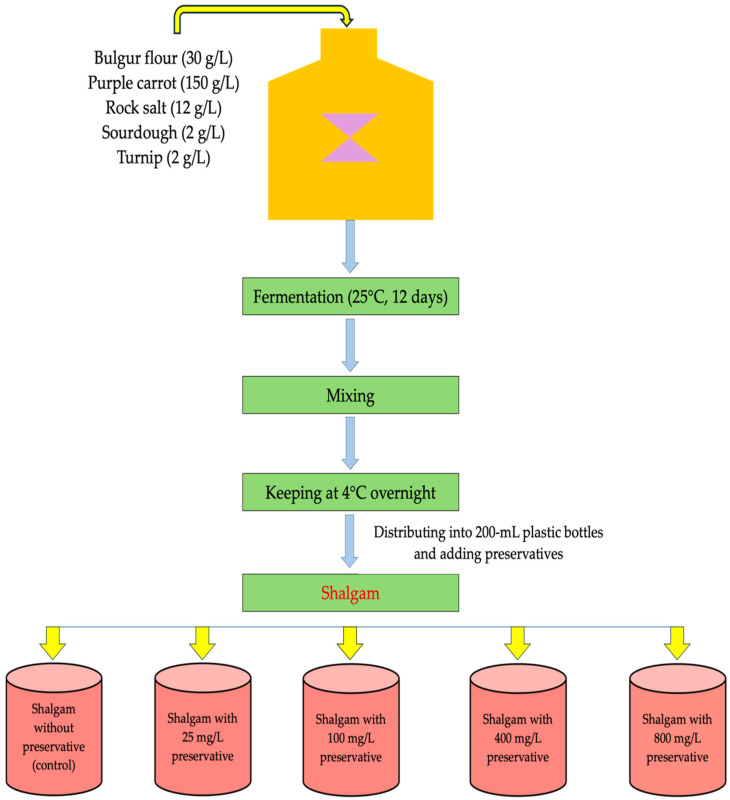
Lab-scale shalgam production diagram.

Shalgam was produced by the direct method according to Tanguler and Erten [3] as shown in Figure 1. After washing purple carrots (150 g/L) and turnips (2 g/L), they were mixed with bulgur flour (30 g/L), sourdough (2 g/L), rock salt (12 g/L), and tap water as required in a 50 L plastic fermentation drum. They were allowed to undergo fermentation in the drum at 25 °C for 12 days. Then, the shalgam juices were homogenized in the drum and transferred to 200 mL plastic bottles after being separated from their residue.

To improve the storage quality of shalgam juices, they were treated individually with three different preservatives: sodium benzoate, potassium sorbate, and natamycin. The preservatives were applied at four different concentrations (25, 100, 400, and 800 mg/L) to shalgam samples that had been fermented and transferred to bottles. In addition, a sample without preservatives was used as a control. All shalgam samples were stored at 4 °C for six months.

### 2.3. Determination of the Physicochemical Properties of Shalgam

The total acidity value was evaluated via titration with sodium hydroxide (0.1 N) until the pH value reached 8.1 in shalgam, and the results were given in terms of lactic acid. pH measurements in shalgam samples were carried out directly with the help of a digital pH meter (VWR International Ltd., Dublin, Ireland) [3]. The total acidity and pH analyses were performed in three replications.

The color of each shalgam sample was measured using a Minolta CR-400 model (Konika Minolta Optics Inc., Tokyo, Japan) brand colorimeter, and the values were found according to the international L*, a*, and b* systems. The results are the average of three replications. The L* value takes variable values between 0 and 100 and gives information about the brightness. In the 3D coordinate system, when it is at 0, the color becomes lighter as it goes towards darkness 100. The a* and b* represent the chromaticity coordinates. The a* value gives different colors; in the positive and negative value ranges, the positive value is red in the color range, and the negative value is green in the color range. The b* value represents yellowness in the positive value range and blueness in the negative value range [20]. 

The results of the physicochemical properties of shalgam samples were evaluated using one-way analysis of variance (ANOVA) and the Tukey multiple comparisons test. *p* < 0.05 values were considered to be significant. In addition, the results were also evaluated using principal component analysis (PCA). The statistical analyses were performed using the Minitab 17 (Minitab Inc., State College, PA, USA) package program.

### 2.4. Enumeration of LAB and Yeasts in Shalgam

Shalgam samples were homogenized in an orbital shaker for 10 sec at room temperature, and their serial dilutions were prepared using 0.85% saline solution. Then, 0.1 mL of diluted samples was spread on de Man, Rogosa, and Sharpe (MRS) Agar (Merck KgaA, Darmstadt, Germany) in Petri dishes via the smear cultivation method. Inoculations were made on four Petri plates from each dilution set. Petri dishes were incubated at 30 °C in anaerobic jars containing oxygen-removing gas packages, Anaerocult^®^ A (Merck KgaA, Darmstadt, Germany), for 2–3 days. LAB counts were determined at the end of incubation [21]. Results were expressed as log colony-forming units (CFU)/mL of the sample.

The yeast counts of shalgam samples were determined by culturing serial dilutions on Potato Dextrose Agar (Merck KgaA, Darmstadt, Germany). Petri dishes were incubated aerobically at 25 °C for 3–4 days [22].

### 2.5. Isolation of LAB

The selected colonies showing different morphologies on MRS agar medium were transferred to MRS broth and incubated at 30 °C for 24 h. For pure culture on slanted MRS agar, different code numbers were given to each isolate. The isolates were coded as PS (potassium sorbate), SB (sodium benzoate), NAT (natamycin), or C (without preservatives, control) based on which preservatives were used in the production of shalgam, followed by a progressive number of isolations.

The LAB isolates were kept at 4 °C on MRS slant agar until identification. The LAB isolates were also stocked at −80 °C in MRS broth containing 15% (*v*/*v*) glycerol.

### 2.6. MALDI-TOF/TOF MS Profile Acquisition

Sample preparation was conducted according to Karasu-Yalcin et al. [18] using the protein extraction method. To extract the cells using this method, 1 mL aliquots of liquid culture were taken and centrifuged at 13,000× *g* for 2 min. The cell pellet was rinsed twice with sterile distilled water and air-dried for 20 min. Following this, the cells were lysed with 70% formic acid (the volume used was proportional to the size of the cell pellet: approximately 30 L), and acetonitrile was added in an equal volume. The supernatant was spotted onto MTP 384 Ground Steel Target (#8280784 Bruker Daltonics, Bremen, Germany) following extensive vortexing and centrifugation (13,000× *g*, 2 min). For each aliquot, a total of four spots for sampling (1 L each) were analyzed. After the sample spot had air-dried, it was covered with 1 L of the matrix (10 mg/mL α-cyano-4-hydroxy-cinnamic acid [α-CHCA], Bruker) and allowed to air-dry once more before being analyzed with the Autoflex Speed (Bruker) using MALDI-TOF MS. The instrument is equipped with a 355 nm nitrogen laser, which was discharged in linear positive mode at the sample spots at a frequency of 55 Hz.

MALDI TOF/TOF MS (Autoflex Speed from Bruker Daltonics, Bremen, Germany) in combination with the MALDI Biotyper 3.1 software program was used for identification based on the analysis of mass spectra. The mass spectrometer was calibrated with a bacterial test standard from Bruker. This calibration kit comprises a typical protein extract of *Escherichia coli* DH5 alpha spiked with two additional pure proteins (RNAse A and myoglobin) to cover an overall mass range of 3.6 to 17 kDa. Before each analysis, the calibration procedure was performed again. MS-signals were acquired for each sample in linear positive mode between 2000 and 20,000 Da *m*/*z* by summing 500 laser-shot spectra in accordance with the manufacturer’s automatic technique, MBT_FC.par. The voltages of the IS1 and IS2 ion sources were 19.99 kV and 19.80 kV, respectively. The lens had a voltage of 6500 kV and an extraction pulse of 200 nanoseconds. The laser intensity was between 50 and 60%.

### 2.7. Identification of LAB by MALDI-TOF/TOF MS

The identification of LAB by MALDI-TOF/TOF MS was conducted in the Scientific, Industrial, and Technological Application and Research Center of Bolu Abant Izzet Baysal University, Turkey. Mass spectra were analyzed using Biotyper software (version 3.1; Bruker Daltonics) and the Biotyper database version DB-6903, which contained 6903 reference MALDI-TOF MS profiles (6120 bacteria, 776 fungi, and 7 archaea). Using a score, the Biotyper software quantified the degree of similarity between experimental profiles obtained from microorganism isolates and reference profiles. The value of the score is determined by the similarity between the observed and stored datasets. A score greater than 2.3 (green) indicates highly probable species-level identification, while a score between 2.0 and 2.3 indicates secure genus identification and probable species identification. A score between 1.7 and 2.0 (yellow) indicates a probable identification of the genus. In contrast, a score value of less than 1.7 (red) indicates that there is no substantial similarity between the unknown profile and the database [18].

## 3. Results and Discussion

### 3.1. Physicochemical Properties of Shalgam

The physicochemical properties of shalgam samples stored at room temperature for six months are summarized in Table 1.

The pH values of shalgam samples varied from 3.43 to 3.52. At the beginning of storage, the total acidity values of all shalgam samples were greater than 7 g/L. These values are in agreement with the shalgam standard (TS 11149) in Turkey [16]. However, total acidy values decreased to 4.41–5.61 g/L after six months of storage. While there was no statistically significant effect of the preservative type and amount used on pH value (*p* > 0.05), its impact on total acidity was found to be significant at a 5% level of significance (*p* < 0.05). The total acidity values of the shalgam samples obtained by adding four different concentrations of each of the three preservatives were found to be higher than those of the control sample. This shows that the decrease in the total acidity amount was higher in the control sample with storage, and the decrease in total acidity remained at a lower level with the increasing amount of preservatives. The highest total acidity (5.61 g/L) was determined in samples treated with sodium benzoate at 100 mg/L, while the lowest value (4.41 g/L) was observed in the control sample.

**Figure 2:**
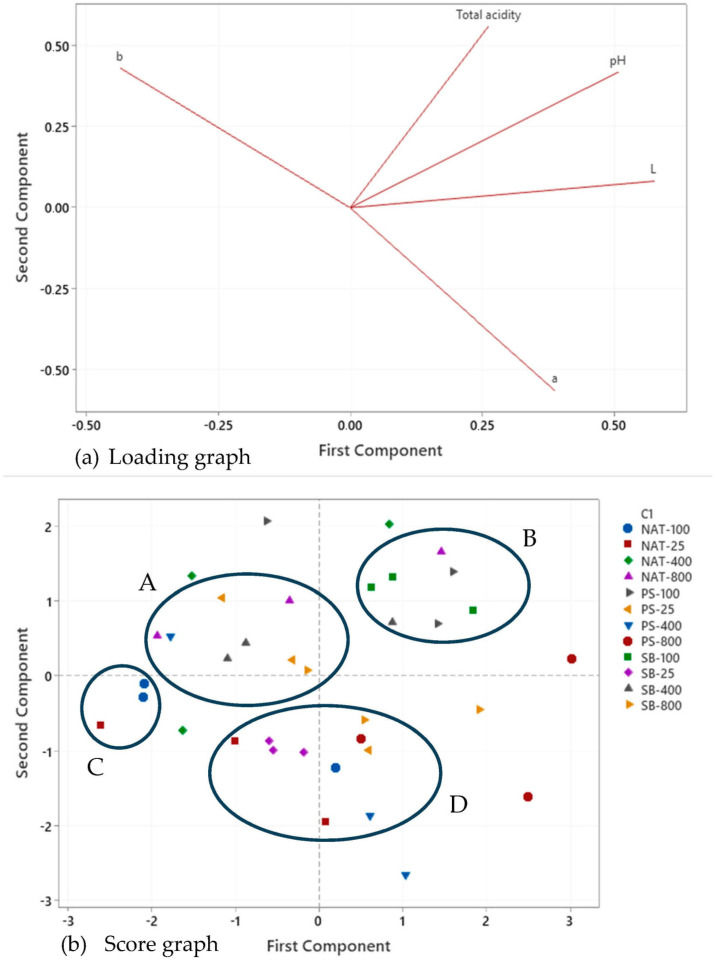
Loading graph (**a**) and score graph (**b**) express the distribution of physicochemical properties of shalgam samples stored at room temperature for six-month samples.

The color of the beverages is one of the important factors influencing consumer preference, and it is one of the most characteristic and significant features of shalgam. While the effects of different preservatives and varying amounts of preservatives on the L* and b* values of shalgam juice were not statistically significant (*p* > 0.05), the impact on the redness value (a*), an important quality criterion for shalgam juice, was found to be significant at a 5% significance level (*p* < 0.05). The L* value was found to be higher than the control sample in all trials using preservatives. Therefore, it can be said that the addition of preservatives caused a slight lightening of the shalgam samples. An increase in L* values and, accordingly, lightening in color values was observed with an increasing amount of preservative concentration. The a* values were positive in shalgam samples treated with preservatives at four different concentrations, ranging from 0.166 to 0.747. In the trials in which preservatives were added, redness generally decreased due to the increased amount of preservative. However, the a* value was −2.467 in the control sample. Since it was determined to have a negative value in the control sample, it can be said that the green color gains weight compared to other samples. In a previous study, L*, a*, and b* values in the shalgams stored for 90 days were found in the range of 4.93–8.84, 26.21–31.46, and 7.72–10.53, respectively [23]. In the trials in which preservatives were added, redness generally decreased depending on the increased amount of preservative. Anthocyanins, which give color to shalgam, can be degraded under the influence of factors such as storage and temperature. Moreover, the active microflora in shalgam during the storage process may also cause a change in acidity, causing a change in the color of the product. The physicochemical data were also applied to the PCA, one of the multivariate analysis methods. The effect of different preservatives on the physicochemical properties of shalgam was compared using a PCA loading graph (Figure 2a) and a score graph (Figure 2b).

The score graph illustrates 2PCs that account for 65.7% of the total variance, leaving approximately 30.3% of unexplained variation. PC1 and PC2 comprised 38.3% and 27.5% of the total variance, respectively. In PC1, a positive correlation was observed with total acidity, L*, a*, and pH, while a negative correlation was found with the b* value. Conversely, in PC2, a negative correlation was identified with the a* value, and a positive correlation was observed with total acidity, L*, b*, and pH values. The relationships between these variables are also depicted in Figure 2a. According to this graph, a strong correlation exists between total acidity and pH, as well as between pH and L* value, as evidenced by the small angles between them.

The PCA reveals a clear separation of samples based on various physicochemical characteristics (Figure 2b). The distance between data points indicates their similarity, with closer points sharing similar properties and those farther apart displaying dissimilar characteristics. In this context, the majority of the isolates can be categorized into four primary groups denoted as A, B, C, and D. However, one replicate each of SB-800, PS-100, PS-400, and two replicates of PS-800 and NAT-400 samples are situated outside these defined groups. Within cluster A, which comprises NAT-800, PS-25, PS-400, SB-400, and SB-800 samples, there are noticeable similarities in their b* values. Conversely, cluster B, represented by PS-100, SB-100, SB-400, and NAT-800 samples, is characterized by positive values along both PC1 and PC2 in the biplot, signifying their commonalities in terms of L*, pH, and total acidity values. Given the critical importance of total acidity for the shelf life of shalgam samples, it becomes apparent that the preservatives and their concentrations used in cluster B, such as PS at 100 mg/L, SB at 100 and 400 mg/L, and NAT at 400 mg/L, are preferable due to their higher total acidity. On the other hand, SB-25 predominantly aligns along the negative axis of PC1, while SB-100 occupies the positive axis of PC1. Notably, SB-100 is characterized by its total acidity, pH, and L* value.

### 3.2. Enumeration of LAB and Yeast in Shalgam Samples

The LAB and total yeast count in shalgam samples treated with different preservatives and stored for six months are given in Table 2.

At the end of the storage period, the LAB and total yeast count in the non-treated shalgam were 6.58 and 6.39 log CFU/mL, respectively. The effect of adding various amounts of different preservatives to shalgam juice, stored for six months, was found to be statistically significant (*p* < 0.05) on LAB and yeasts. As expected, the preservative treatment resulted in 2-log or greater reductions in LAB and yeast in shalgam. The counts of LAB and yeast in shalgam treated with preservatives varied from 2.48 to 4.60 log CFU/mL and from 1.31 to 4.01 log CFU/mL, respectively. It has been observed that the counts of LAB and yeast decreased with an increase in the concentration of preservatives. In a previous report by Tanguler [24] LAB and yeast counts were found in the range of 7.04–7.60 log CFU/mL and 5.79–6.62 log CFU/mL in shalgam samples, respectively. In another study, the numbers of LAB and yeast were found to be 7.40 and 2.62 log CFU/mL in shalgam samples stored for 3 months, respectively [25]. In shalgam samples stored for a month, the LAB count was 8.89 log CFU/mL and the yeast count was 8.79 log CFU/mL [8].

The results indicated that sodium benzoate causes a yeast reduction of 2 to 5 log CFU/mL, with the greatest reduction at the highest concentration of the preservative (800 mg/L). Yeasts can grow in an acidic environment and may cause undesirable results such as film formation, color changes, and taste deterioration in shalgam [26]. In Turkey, sodium benzoate is widely used in shalgam during production to extend shelf life by suppressing spoilage yeast and mold [27]. The maximum allowable concentration of benzoates in shalgam is set at 200 mg/L [16].

Natamycin treatment also resulted in large reductions (3–5 log CFU/mL) in the yeast population. As expected, it was less effective on LAB; a 2–3 log CFU/mL reduction was observed in the LAB population. There was no significant difference in LAB reduction between the treatments with different natamycin concentrations except for 25 mg/L.

The use of potassium sorbate at a concentration of 100 mg/L reduced the yeast count by approximately 3 log units. The use of over 100 mg/L was not sensible for this study, as it did not cause a significant change in the yeast count. The results showed that the use of potassium sorbate up to 100 mg/L can be an alternative to benzoates (100 mg/L).

### 3.3. Identification of LAB by MALDI-TOF/TOF MS

In total, 100 isolates were collected from control and preservative-treated shalgam samples. The isolates’ MALDI-TOF/TOF MS profiles were identified by comparing them to BioTyper’s reference spectra. The isolate codes, sources, and MALDI-TOF/TOF MS identification results are presented in Table 3.

**Figure 3:**
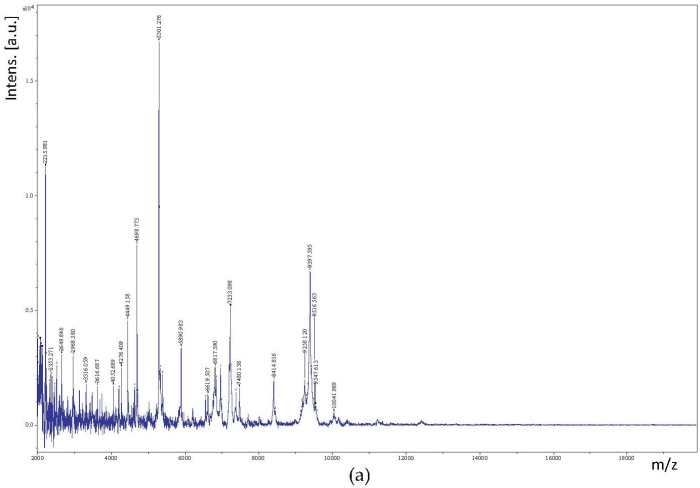

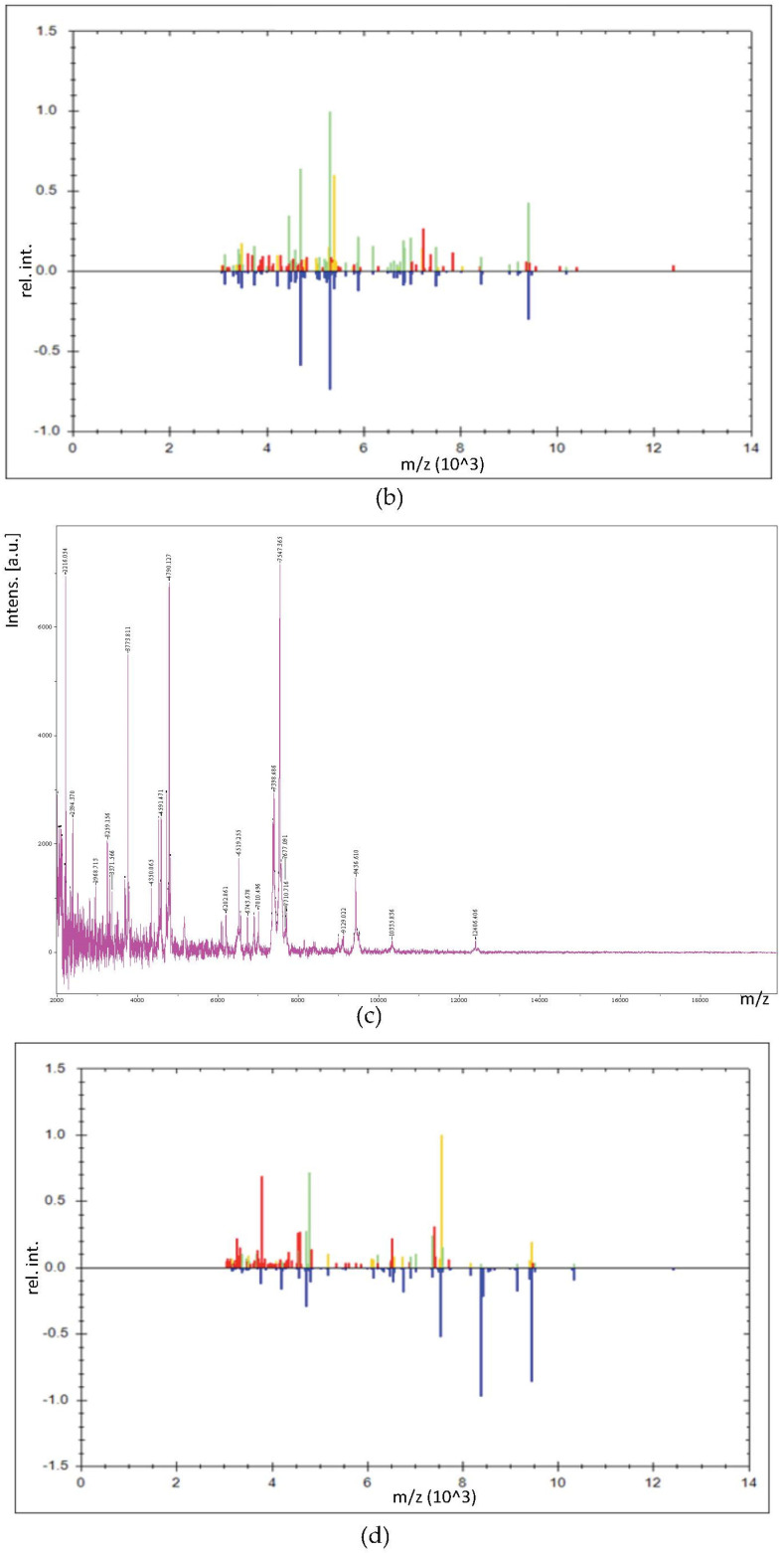

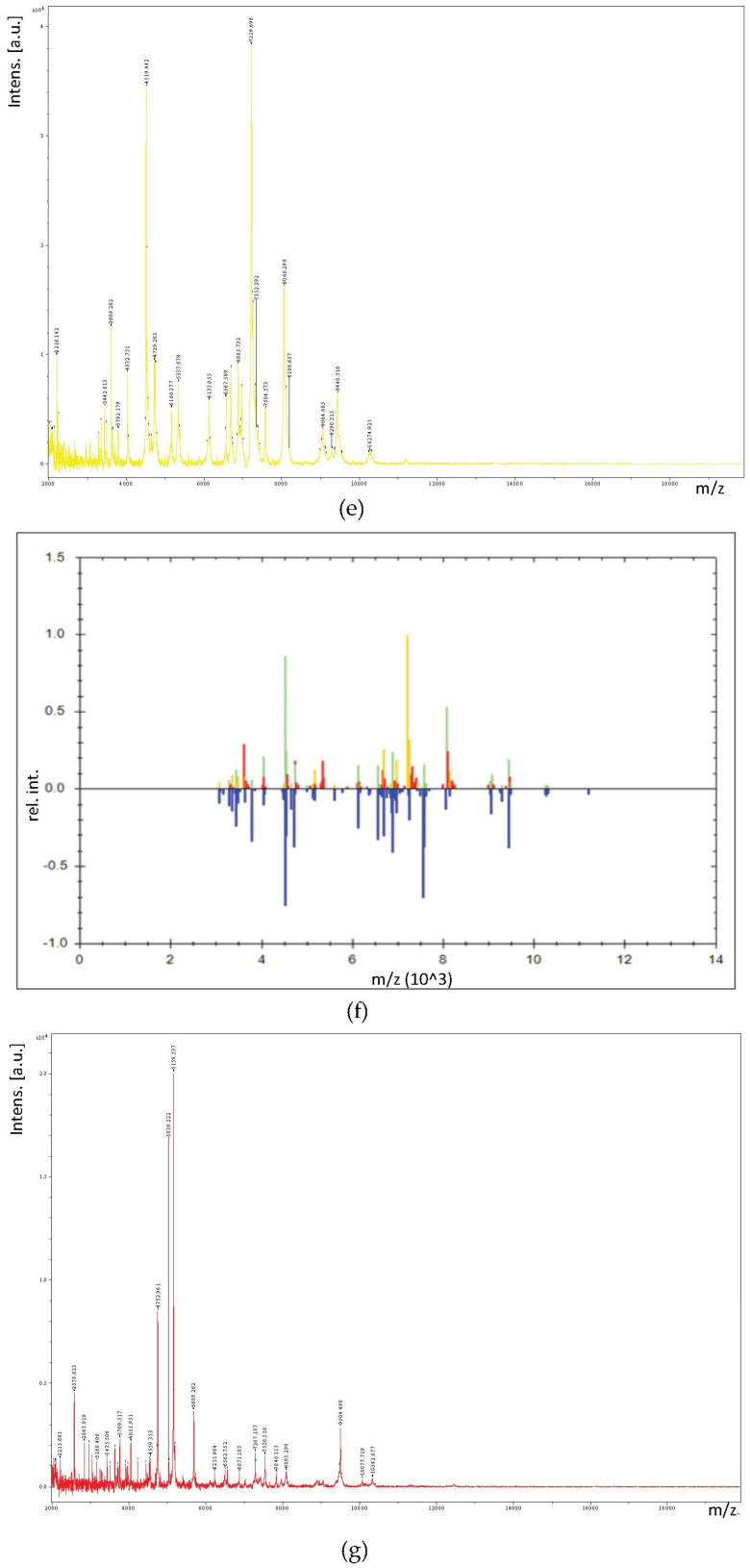

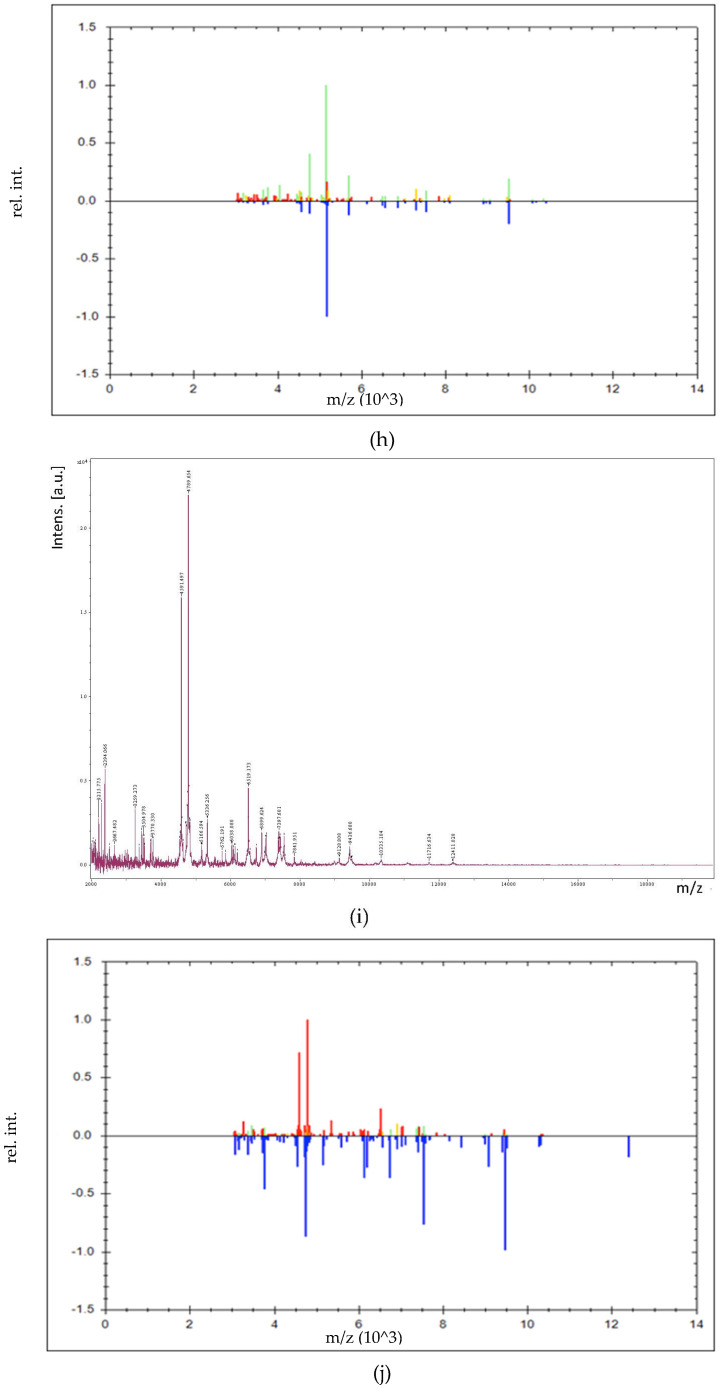

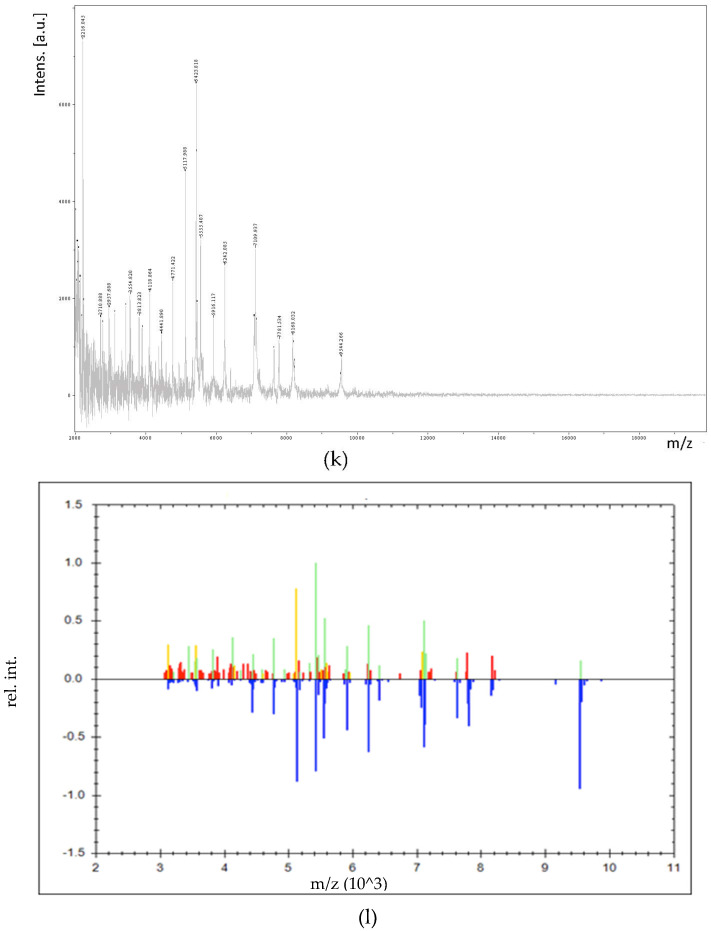
(**a**,**c**,**e**,**g**,**i**,**k**): raw MALDI-TOF MS profile of *Lacticaseibacillus paracasei* subsp. *paracasei*, *Lentilactobacillus buchneri*, *Levilactobacillus brevis*, *Lactiplantibacillus pentosus*, *Lentilactobacillus kefiri*, and *Leuconostoc mesenteroides*, respectively. (**b**,**d**,**f**,**h**,**j**,**l**): matching results of experimental profiles of *Lacticaseibacillus paracasei* subsp. *paracasei*, *Lentilactobacillus buchneri*, *Levilactobacillus brevis*, *Lactiplantibacillus pentosus*, *Lentilactobacillus kefiri*, and *Leuconostoc mesenteroides* and the BioTyper database, respectively.

Of the 100 isolates, MALDI-TOF/TOF MS was able to identify 89 isolates with high confidence. The isolates identified by this method belonged to the species *Lacticaseibacillus paracasei* subsp. *paracasei*, *Lentilactobacillus* (*buchneri*, *Levilactobacillus brevis*, *Lactiplantibacillus pentosus*, *Lentilactobacillus kefiri*, and *Leuconostoc mesenteroides*. The mass spectra of *Lacticaseibacillus paracasei* subsp. *paracasei*, *Lentilactobacillus buchneri*, *Levilactobacillus brevis*, *Lactiplantibacillus pentosus*, *Lentilactobacillus kefiri*, and *Leuconostoc mesenteroides* species are shown in Figure 3. Almost all identified strains matched with score values near or higher than 2.3, which indicates highly probable species-level identification.

**Figure 4:**
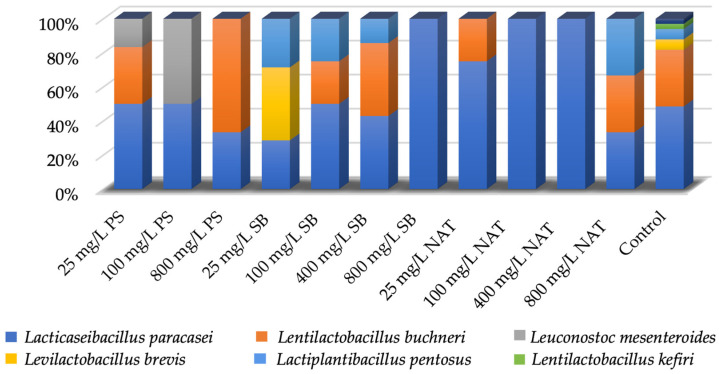
Percentage distribution of isolates obtained from non-treated and preservative-treated shalgam at four different concentrations.

The species-specific percentage distribution of isolates obtained from non-treated (control) and preservative-treated shalgam samples at different concentrations is depicted in Figure 4. A total of 32 LAB isolates were identified from unpreserved shalgam samples. These isolates were identified as belonging to the species *Lacticaseibacillus paracasei* subsp. *paracasei* (*n* = 16), *Levilactobacillus brevis* (*n* = 2), *Lentilactobacillus buchneri* (*n* = 11), *Lactiplantibacillus pentosus* (*n* = 2), and *Lentilactobacillus kefiri* (*n* = 1). *Lacticaseibacillus paracasei* subsp. *paracasei* was found to be the predominant LAB species in the shalgam samples analyzed, and the use of various concentrations of preservatives did not affect this result. The use of preservatives reduced the diversity of LAB in shalgam samples compared to the control samples.

In contrast to control samples, strains of the *Leuconostoc mesenteroides* species (PS-6, PS-8, and PS-10) were isolated from shalgam samples in experiments in which potassium sorbate was used as a preservative. In addition, *Lacticaseibacillus paracasei* subsp. *paracasei* and *Lentilactobacillus buchneri* species were identified among the isolates. However, potassium sorbate was found to inhibit the growth of the *Levilactobacillus brevis*, *Lactiplantibacillus pentosus*, and *Lentilactobacillus kefiri* species. While *Leuconostoc mesenteroides* was not detected in potassium sorbate-treated shalgam samples at 800 mg/L, it was found in shalgam samples treated with potassium sorbate at lower concentrations. Figure 4 demonstrates that when 25 mg/L potassium sorbate was used, 50% of the isolated bacteria belonged to *Lacticaseibacillus paracasei* subsp. *paracasei*, 33% to *Lentilactobacillus buchneri*, and 17% to *Leuconostoc mesenteroides*. In shalgam samples treated with potassium sorbate at 100 mg/L, 50% of the isolates were *Lacticaseibacillus paracasei* subsp. *paracasei*, and the remaining 50% were *Leuconostoc mesenteroides*. However, *Lentilactobacillus buchneri* was the main species isolated from potassium sorbate-treated shalgam at 800 mg/L.

A total of 33 strains isolated from shalgam samples, in which sodium benzoate was used as a preservative, could be identified (Table 3). Among them, strains SB-1, SB-5, and SB-6, belonging to the *Levilactobacillus brevis* species, were isolated only when 25 mg/L of sodium benzoate was used. When 100 mg/L sodium benzoate was used, 50% of the isolated strains were *Lacticaseibacillus paracasei* subsp. *paracasei*, 25% *Lentilactobacillus buchneri*, and 25% *Lactiplantibacillus pentosus* (Figure 4). These three species were also isolated from shalgam treated with sodium benzoate at 400 and 800 mg/L.

*Lacticaseibacillus paracasei* subsp. *paracasei* was the predominant species in shalgam treated with natamycin at 25, 100, and 400 mg/L. When the natamycin concentration was 800 mg/L, it was determined that *Lacticaseibacillus paracasei* subsp. *paracasei*, *Lentilactobacillus buchneri*, and *Lactiplantibacillus pentosus* species showed an equal distribution.

Lactic acid bacteria play an important role in the formation of shalgam-specific taste and odor; therefore, it is important to determine the flora of shalgam produced via the traditional spontaneous fermentation method. Previous studies have demonstrated the prevalence of *Lactobacillus* species in shalgam [3,28]. Tanguler and Erten [3] emphasized *Lactobacillus*. *paracasei* subsp. *paracasei* (new name: *Lacticaseibacillus paracasei* subsp. *paracasei*) is the most important LAB bacteria species quantitatively among the LAB that were isolated during shalgam fermentation. In another study, *Lactobacillus buchneri* (new name: *Lentilactobacillus buchneri*), *Lactobacillus casei* (new name: *Lacticaseibacillus casei*), *Lactobacillus brevis* (new name: *Levilactobacillus brevis*), and *Lactobacillus plantarum* (new name: *Lactiplantibacillus plantarum*) were defined at the end of the fermentation process in shalgams produced using the direct method. However, *Leuconostoc mesenteroides*, which was initially isolated at the beginning of the fermentation, could not be isolated at the end of the fermentation process [29]. 

During the fermentation of shalgam, *Lactobacillus plantarum* was found to be the most common strain. However, other LAB, such as *Lactobacillus brevis*, *Lactococcus lactis*, and *Leuconostoc mesenteroides*, were also found [30]. *Lactobacillus plantarum* was also discovered to be the predominant species of LAB during shalgam fermentation in a previous work by Erginkaya and Turhan [31]. Low numbers of *Lactobacillus pentosus* (new name: *Lactiplantibacillus pentosus*) were also isolated. Unfortunately, *Lactiplantibacillus plantarum*, which was determined to be the dominant species in previous studies [24,29,30], could not be detected in shalgam samples with neither a non-treated nor a preservative added in our study. *Lactiplantibacillus plantarum* has probiotic potential with tolerance to the gastrointestinal environment and colonization in the gut [32]. On the other hand, in the present study, *Lactiplantibacillus pentosus* was isolated from non-treated and preservative-treated shalgam samples. It seems likely that this is the first time *Lentilactobacillus kefiri* has been detected in shalgam. Additionally, it is highlighted that *Lentilactobacillus kefiri* was only isolated from the non-treated shalgam samples, indicating that it was unable to withstand the presence of preservative chemicals.

## 4. Conclusions

This study has focused on the effects of sodium benzoate, potassium sorbate, and natamycin on the physicochemical and microbiological properties of shalgam stored at room temperature for six months. The preservative additives had no discernible impact on the physicochemical properties of shalgam, but they did significantly reduce the number of yeasts with deterioration potential. A total of 89 LAB isolated from non-treated and preservative-treated shalgam samples were identified with high confidence using the MALDI-TOF/TOF MS method. *Lacticaseibacillus paracasei* subsp. *paracasei*, *Levilactobacillus brevis*, *Lentilactobacillus buchneri*, *Lactiplantibacillus pentosus*, and *Lentilactobacillus kefiri* species were isolated from non-treated shalgam. Different LAB species were found in shalgam that had been treated with preservatives, depending on the type and amount of preservatives. However, *Lacticaseibacillus paracasei* subsp. *paracasei* could be found in almost all cases. It is known that the *Lacticaseibacillus paracasei* isolated from fermented products has probiotic properties and therefore promotes human health. This study reveals that preservative additives extend the shelf life of shalgam; however, they can lead to a reduction in the population of LAB, especially at high concentrations. It can be concluded that the most effective approach to shalgam production results in the highest total acidity value when sodium benzoate is employed at a concentration of 100 mg/L.

## Figures and Tables

**Table 1:** The physicochemical properties of shalgam samples stored at room temperature for six months. Values are expressed as mean ± standard deviation (SD) of *n* = 3 samples.

Samples	pH	Total Acididy (as Lactic Acid g/L)	Instrumental Color Parameters		
			L*	a*	b*
Control	3.43 ± 0.01	4.41 ± 0.13 ^e^	23.97 ± 1.67	−2.467 ± 0.70 ^b^	−4.387 ± 1.22
SB-25	3.46 ± 0.01	5.13 ± 0.04 ^bcd^	24.88 ± 0.46	0.525 ± 0.01 ^a^	−4.433 ± 0.22
SB-100	3.52 ± 0.03	5.61 ± 0.13 ^a^	26.05 ± 1.44	0.448 ± 0.09 ^a^	−4.436 ± 0.22
SB-400	3.45 ± 0.04	5.37 ± 0.04 ^abcd^	26.97 ± 2.40	0.368 ± 0.09 ^a^	−3.979 ± 0.22
SB-800	3.45 ± 0.01	5.46 ± 0.08 ^abc^	27.20 ± 1.87	0.608 ± 0.09 ^a^	−4.502 ± 0.24
NAT-25	3.44 ± 0.03	4.95 ± 0.13 ^d^	24.90 ± 2.36	0.441 ± 0.09 ^a^	−4.213 ± 0.48
NAT-100	3.45 ± 0.01	5.04 ± 0.17 ^cd^	24.35 ± 2.83	0.304 ± 0.12 ^a^	−4.220 ± 0.61
NAT-400	3.46 ± 0.09	5.31 ± 0.13 ^abcd^	26.29 ± 2.86	0.167 ± 0.17 ^a^	−3.988 ± 0.29
NAT-800	3.49 ± 0.11	5.31 ± 0.13 ^abcd^	26.44 ± 2.69	0.166 ± 0.10 ^a^	−4.098 ± 0.32
PS-25	3.47 ± 0.01	5.27 ± 0.02 ^abcd^	26.09 ± 1.37	0.559 ± 0.32 ^a^	−3.827 ± 0.50
PS-100	3.51 ± 0.01	5.58 ± 0.08 ^ab^	26.86 ± 1.48	0.445 ± 0.23 ^a^	−4.028 ± 0.38
PS-400	3.44 ± 0.01	5.04 ± 0.17 ^cd^	27.29 ± 1.57	0.669 ± 0.45 ^a^	−4.182 ± 0.67
PS-800	3.50 ± 0.12	5.34 ± 0.17 ^abcd^	28.12 ± 1.33	0.747 ± 0.05 ^a^	−4.818 ± 0.64

SB: sodium benzoate. NAT: natamycin. PS: potassium sorbate. The difference between the values shown in different lower-case letters in the same column is statistically different (*p* < 0.05).

**Table 2:** The counts of LAB and yeast in shalgam treated with preservatives and stored at room temperature for six months (mean ± SD).

Treatment	LAB (log CFU/mL)	Yeast (log CFU/mL)
Control	6.58 ± 0.1 ^a^	6.39 ± 0.3 ^a^
SB-25	4.60 ± 0.4 ^ab^	4.01 ± 0.3 ^b^
SB-100	3.03 ± 0.4 ^b^	3.12 ± 0.3 ^bcd^
SB-400	2.65 ± 0.5 ^b^	2.42 ± 0.3 ^def^
SB-800	2.48 ± 1.3 ^b^	1.31 ± 0.7 ^f^
NAT-25	4.55 ± 0.6 ^ab^	3.12 ± 0.2 ^bcd^
NAT-100	3.95 ± 0.9 ^b^	2.92 ± 0.1 ^bcd^
NAT-400	4.03 ± 0.7 ^b^	2.96 ± 0.4 ^bcd^
NAT-800	3.58 ± 0.5 ^b^	1.57 ± 0.2 ^ef^
PS-25	3.93 ± 0.3 ^b^	3.63 ± 0.2 ^bc^
PS-100	3.83 ± 0.1 ^b^	3.39 ± 0.2 ^bcd^
PS-400	3.05 ± 0.3 ^b^	3.19 ± 0.3 ^bcd^
PS-800	2.78 ± 0.8 ^b^	2.52 ± 0.3 ^cde^

SB: sodium benzoate. NAT: natamycin. PS: potassium sorbate. The difference between the values shown in different lower-case letters in the same column is statistically different (*p* < 0.05).

**Table 3:** The isolate codes, sources and MALDI-TOF/TOF MS identification results.

Isolate No	Isolate Code	Isolation Source	Identification Result
1	C-1	Non-treated shalgam	*Lacticaseibacillus paracasei* subsp. *paracasei*
2	C-2	Non-treated shalgam	*Levilactobacillus brevis*
3	C-3	Non-treated shalgam	*Lacticaseibacillus paracasei* subsp. *paracasei*
4	C-4	Non-treated shalgam	*Lentilactobacillus buchneri*
5	C-5	Non-treated shalgam	*Lacticaseibacillus paracasei* subsp. *paracasei*
6	C-6	Non-treated shalgam	*Lacticaseibacillus paracasei* subsp. *paracasei*
7	C-7	Non-treated shalgam	*Lentilactobacillus buchneri*
8	C-8	Non-treated shalgam	*Lactiplantibacillus pentosus*
9	C-9	Non-treated shalgam	*Lacticaseibacillus paracasei* subsp. *paracasei*
10	C-10	Non-treated shalgam	*Lentilactobacillus buchneri*
11	C-11	Non-treated shalgam	*Lacticaseibacillus paracasei* subsp. *paracasei*
12	C-12	Non-treated shalgam	*Lentilactobacillus buchneri*
13	C-13	Non-treated shalgam	*Lacticaseibacillus paracasei* subsp. *paracasei*
14	C-14	Non-treated shalgam	*Lacticaseibacillus paracasei* subsp. *paracasei*
15	C-15	Non-treated shalgam	*Lacticaseibacillus paracasei* subsp. *paracasei*
16	C-16	Non-treated shalgam	*Lacticaseibacillus paracasei* subsp. *paracasei*
17	C-17	Non-treated shalgam	*Lentilactobacillus buchneri*
18	C-18	Non-treated shalgam	*Lacticaseibacillus paracasei* subsp. *paracasei*
19	C-19	Non-treated shalgam	*Lentilactobacillus buchneri*
20	C-20	Non-treated shalgam	*Lentilactobacillus kefiri*
21	C-21	Non-treated shalgam	*Lacticaseibacillus paracasei* subsp. *paracasei*
22	C-22	Non-treated shalgam	*Lactiplantibacillus pentosus*
23	C-23	Non-treated shalgam	*Lentilactobacillus buchneri*
24	C-24	Non-treated shalgam	*Lentilactobacillus buchneri*
25	C-25	Non-treated shalgam	*Lacticaseibacillus paracasei* subsp. *paracasei*
26	C-26	Non-treated shalgam	*Lacticaseibacillus paracasei* subsp. *paracasei*
27	C-27	Non-treated shalgam	*Lentilactobacillus buchneri*
28	C-28	Non-treated shalgam	*Levilactobacillus brevis*
29	C-29	Non-treated shalgam	*Lacticaseibacillus paracasei* subsp. *paracasei*
30	C-30	Non-treated shalgam	*Lentilactobacillus buchneri*
31	C-31	Non-treated shalgam	*Lentilactobacillus buchneri*
32	C-32	Non-treated shalgam	*Lentilactobacillus kefiri*
33	PS-1	Shalgam containing 25 mg/L PS	*Lacticaseibacillus paracasei* subsp. *paracasei*
34	PS-2	Shalgam containing 25 mg/L PS	*Lentilactobacillus buchneri*
35	PS-3	Shalgam containing 25 mg/L PS	*Lacticaseibacillus paracasei* subsp. *paracasei*
36	PS-4	Shalgam containing 25 mg/L PS	*Lacticaseibacillus paracasei* subsp. *paracasei*
37	PS-5	Shalgam containing 25 mg/L PS	*Lentilactobacillus buchneri*
38	PS-6	Shalgam containing 25 mg/L PS	*Leuconostoc mesenteroides*
39	PS-7	Shalgam containing 100 mg/L PS	*Lacticaseibacillus paracasei* subsp. *paracasei*
40	PS-8	Shalgam containing 100 mg/L PS	*Leuconostoc mesenteroides*
41	PS-9	Shalgam containing 100 mg/L PS	*Lacticaseibacillus paracasei* subsp. *paracasei*
42	PS-10	Shalgam containing 100 mg/L PS	*Leuconostoc mesenteroides*
43	PS-11	Shalgam containing 800 mg/L PS	*Lentilactobacillus buchneri*
44	PS-12	Shalgam containing 800 mg/L PS	*Lacticaseibacillus paracasei* subsp. *paracasei*
45	PS-13	Shalgam containing 800 mg/L PS	*Lentilactobacillus buchneri*
46	SB-1	Shalgam containing 25 mg/L SB	*Levilactobacillus brevis*
47	SB-2	Shalgam containing 25 mg/L SB	*Lacticaseibacillus paracasei* subsp. *paracasei*
48	SB-3	Shalgam containing 25 mg/L SB	*Lactiplantibacillus pentosus*
49	SB-4	Shalgam containing 25 mg/L SB	*Lacticaseibacillus paracasei* subsp. *paracasei*
50	SB-5	Shalgam containing 25 mg/L SB	*Levilactobacillus brevis*
51	SB-6	Shalgam containing 25 mg/L SB	*Levilactobacillus brevis*
52	SB-7	Shalgam containing 25 mg/L SB	*Lactiplantibacillus pentosus*
53	SB-8	Shalgam containing 25 mg/L SB	*Lacticaseibacillus paracasei* subsp. *paracasei*
54	SB-9	Shalgam containing 100 mg/L SB	*Lacticaseibacillus paracasei* subsp. *paracasei*
55	SB-10	Shalgam containing 100 mg/L SB	*Lactiplantibacillus pentosus*
56	SB-11	Shalgam containing 100 mg/L SB	*Lacticaseibacillus paracasei* subsp. *paracasei*
57	SB-12	Shalgam containing 100 mg/L SB	*Lacticaseibacillus paracasei* subsp. *paracasei*
58	SB-13	Shalgam containing 100 mg/L SB	*Lentilactobacillus buchneri*
59	SB-14	Shalgam containing 100 mg/L SB	*Lactiplantibacillus pentosus*
60	SB-15	Shalgam containing 100 mg/L SB	*Lacticaseibacillus paracasei* subsp. *paracasei*
61	SB-16	Shalgam containing 100 mg/L SB	*Lentilactobacillus buchneri*
62	SB-17	Shalgam containing 400 mg/L SB	*Lacticaseibacillus paracasei* subsp. *paracasei*
63	SB-18	Shalgam containing 400 mg/L SB	*Lentilactobacillus buchneri*
64	SB-19	Shalgam containing 400 mg/L SB	*Lentilactobacillus buchneri*
65	SB-20	Shalgam containing 400 mg/L SB	*Lacticaseibacillus paracasei* subsp. *paracasei*
66	SB-21	Shalgam containing 400 mg/L SB	*Lentilactobacillus buchneri*
67	SB-22	Shalgam containing 400 mg/L SB	*Lacticaseibacillus paracasei* subsp. *paracasei*
68	SB-23	Shalgam containing 400 mg/L SB	*Lentilactobacillus buchneri*
69	SB-24	Shalgam containing 400 mg/L SB	*Lacticaseibacillus paracasei* subsp. *paracasei*
70	SB-25	Shalgam containing 400 mg/L SB	*Lactiplantibacillus pentosus*
71	SB-26	Shalgam containing 400 mg/L SB	*Lacticaseibacillus paracasei* subsp. *paracasei*
72	SB-27	Shalgam containing 400 mg/L SB	*Lentilactobacillus buchneri*
73	SB-28	Shalgam containing 400 mg/L SB	*Lacticaseibacillus paracasei* subsp. *paracasei*
74	SB-29	Shalgam containing 400 mg/L SB	*Lactiplantibacillus pentosus*
75	SB-30	Shalgam containing 400 mg/L SB	*Lentilactobacillus buchneri*
76	SB-31	Shalgam containing 800 mg/L SB	*Lacticaseibacillus paracasei* subsp. *paracasei*
77	SB-32	Shalgam containing 800 mg/L SB	*Lentilactobacillus buchneri*
78	SB-33	Shalgam containing 800 mg/L SB	*Lacticaseibacillus paracasei* subsp. *paracasei*
79	NAT-1	Shalgam containing 25 mg/L NAT	*Lentilactobacillus buchneri*
80	NAT-2	Shalgam containing 25 mg/L NAT	*Lacticaseibacillus paracasei* subsp. *paracasei*
81	NAT-3	Shalgam containing 25 mg/L NAT	*Lacticaseibacillus paracasei* subsp. *paracasei*
82	NAT-4	Shalgam containing 25 mg/L NAT	*Lacticaseibacillus paracasei* subsp. *paracasei*
83	NAT-5	Shalgam containing 100 mg/L NAT	*Lacticaseibacillus paracasei* subsp. *paracasei*
84	NAT-6	Shalgam containing 100 mg/L NAT	*Lacticaseibacillus paracasei* subsp. *paracasei*
85	NAT-7	Shalgam containing 100 mg/L NAT	*Lacticaseibacillus paracasei* subsp. *paracasei*
86	NAT-8	Shalgam containing 400 mg/L NAT	*Lacticaseibacillus paracasei* subsp. *paracasei*
87	NAT-9	Shalgam containing 800 mg/L NAT	*Lacticaseibacillus paracasei* subsp. *paracasei*
88	NAT-10	Shalgam containing 800 mg/L NAT	*Lentilactobacillus buchneri*
89	NAT-11	Shalgam containing 800 mg/L NAT	*Lactiplantibacillus pentosus*

C: control, non-treated. PS: potassium sorbate. SB: sodium benzoate. NAT: natamycin.

## Data Availability

The data used to support the findings of this study can be made available by the corresponding author upon request.

## References

[1] Behera S.S., El Sheikha A.F., Hammami R., Kumar A. (2020). Traditionally fermented pickles: How the microbial diversity associated with their nutritional and health benefits?. J. Funct. Foods.

[2] Cuamatzin-García L., Rodríguez-Rugarcía P., El-Kassis E.G., Galicia G., Meza-Jiménez M.D.L., Baños-Lara M.D.R., Zaragoza-Maldonado D.S., Pérez-Armendáriz B. (2022). Traditional fermented foods and beverages from around the world and their health benefits. Microorganisms.

[3] Tanguler H., Erten H. (2012). Occurrence and growth of lactic acid bacteria species during the fermentation of shalgam (salgam), a traditional Turkish fermented beverage. LWT-Food Sci. Technol..

[4] Gok I. (2023). Functional potential of several Turkish fermented traditional foods: Biotic properties, bioactive compounds, and health benefits. Food Rev. Int..

[5] Kırlangıç O., Ilgaz C., Kadiroğlu P. (2021). Influence of pasteurization and storage conditions on microbiological quality and aroma profiles of shalgam. Food Biosci..

[6] Karaoglan H.A., Keklik N.M., Develi Işikli N. (2017). Modeling inactivation of *Candida inconspicua* isolated from turnip juice using pulsed UV light. J. Food Process. Eng..

[7] Coskun F. (2017). A traditional Turkish fermented non-alcoholic beverage, “Shalgam”. Beverages.

[8] Yanardağ Karabulut Ş. (2020). Glutensiz ve Katkısız Şalgam Suyu Üretimi ve Yüksek Hidrostatik Basınç ile raf Ömrünün Uzatılması. Master’s Thesis.

[9] Ulucan E., Çoklar H., Akbulut M. (2022). Application of ultrasound to extend the shelf life of the shalgam juice: Changes in various physicochemical, nutritional, and microbiological properties. J. Food Process. Preserv..

[10] Silva M.M., Lidon F.C. (2016). Food preservatives—An overview on applications and side effects. Emir. J. Food Agric..

[11] Hoca G. (2019). Bursa Ilinde Tüketime Sunulan nar Ekşisi ve Nar Ekşili Soslarda Sorbik asit ve Benzoik Asit Miktarlarının Belirlenmesi. Master’s Thesis.

[12] Piper J.D., Piper P.W. (2017). Benzoate and sorbate salts: A systematic review of the potential hazards of these invaluable preservatives and the expanding spectrum of clinical uses for sodium benzoate. Comr. Rev. Food Sci. Food Saf..

[13] Piper P.W. (2018). Potential safety issues surrounding the use of benzoate preservatives. Beverages.

[14] Stark J., Robinson R.K. (1999). Preservatives/permitted preservatives-natamycin. Encyclopedia of Food Microbiology.

[15] Lule V.K., Garg S., Gosewade S.C., Khedkar C.D., Caballero B., Finglas P.M., Toldrá F. (2016). Natamycin. Encyclopedia of Food and Health.

[16] (2016). Turnip Juice.

[17] Topić Popović N., Kazazić S.P., Bojanić K., Strunjak-Perović I., Čož-Rakovac R. (2023). Sample preparation and culture condition effects on MALDI-TOF MS identification of bacteria: A review. Mass Spectrom. Rev..

[18] Karasu-Yalcin S., Soylemez-Milli N., Eren O., Eryasar-Orer K. (2021). Reducing time in detection of *Listeria monocytogenes* from food by MALDI-TOF mass spectrometry. J. Food Sci. Technol..

[19] Singhal N., Kumar M., Kanaujia P.K., Virdi J.S. (2015). MALDI-TOF mass spectrometry: An emerging technology for microbial identification and diagnosis. Front. Microbiol..

[20] Hunter R., Hunter R., Harold R.V. (1975). Scales for the measurements of color difference. The Measurements of the Appearance.

[21] Tamang J.P., Tamang B., Schillinger U., Franz C.M.A.P., Gores M., Holzapfel W.H. (2005). Identification of predominant lactic acid bacteria isolated from traditionally fermented vegetable products of the Eastern Himalayas. Int. J. Food Microbiol..

[22] Yusuf D., Nuraida L., Dewanti-Hariyadi R., Hunaefi D. (2020). Lactic acid bacteria and yeasts from Indonesian kefir grains and their growth interaction. Asian J. Microbiol. Biotechnol. Environ. Sci..

[23] Ateş C. (2019). Effectiveness of Ultrasonication and High Pressure Processing Pasteurization on Quality Characteristics and Shelf Life of Fermented Shalgam Drink. Master’s Thesis.

[24] Tangüler H. (2010). Identification of Predominant Lactic acid Bacteria Isolated from Shalgam Beverage and Improvement of Its Production Technique. Ph.D. Thesis.

[25] Ulucan E. (2019). Effects of Some Physicochemical and Microbiological Properties of Ultrasonic Application on Shalgam Juice after Fermentation. Master’s Thesis.

[26] Altay F., Karbancioglu-Güler F., Daskaya-Dikmen C., Heperkan D. (2013). A review on traditional Turkish fermented non-alcoholic beverages: Microbiota, fermentation process and quality characteristics. Int. J. Food Microbiol..

[27] Şanlibaba P., Tezel B.U. (2023). Traditional fermented foods in Anatolia. Acta Sci. Pol. Technol. Aliment..

[28] Mete A., Coşansu S., Demirkol O., Ayhan K. (2017). Amino acid decarboxylase activities and biogenic amine formation abilities of lactic acid bacteria isolated from shalgam. Int. J. Food Prop..

[29] Tanguler H., Saris P.E.J., Erten H. (2015). Microbial, chemical and sensory properties of shalgams made using different production methods. J. Sci. Food Agric..

[30] Kafkasgiray E.S. (2020). Determination of the Microbial Profile of Shalgam Beverage Fermentation Process by Molecular Methods. Master’s Thesis.

[31] Erginkaya Z., Ünal Turhan E. (2016). Enumeration and identification of dominant microflora during the fermentation of shalgam. Akademik Gıda.

[32] Bu Y., Liu Y., Liu Y., Wang S., Liu Q., Hao H., Yi H. (2022). Screening and probiotic potential evaluation of bacteriocin-producing *Lactiplantibacillus plantarum* in vitro. Foods.

